# Programmed Cell Death-Like and Accompanying Release of Microcystin in Freshwater Bloom-Forming Cyanobacterium *Microcystis*: From Identification to Ecological Relevance

**DOI:** 10.3390/toxins11120706

**Published:** 2019-12-04

**Authors:** Chenlin Hu, Piotr Rzymski

**Affiliations:** 1 College of Pharmacy, University of Houston, Houston, TX 77204, USA; 2 Department of Environmental Medicine, Poznan University of Medical Sciences, 60-806 Poznan, Poland

**Keywords:** cyanobacteria, *Microcystis*, programmed cell death, apoptosis, microcystin

## Abstract

*Microcystis* is the most common freshwater bloom-forming cyanobacterium. Its massive blooms not only adversely affect the functionality of aquatic ecosystems, but are also associated with the production of microcystins (MCs), a group of potent toxins that become a threat to public health when cell-bound MCs are significantly released from the dying *Microcystis* into the water column. Managing *Microcystis* blooms thus requires sufficient knowledge regarding both the cell death modes and the release of toxins. Recently, more and more studies have demonstrated the occurrence of programmed cell death-like (or apoptosis-like) events in laboratory and field samples of *Microcystis*. Apoptosis is a genetically controlled process that is essential for the development and survival of metazoa; however, it has been gradually realized to be an existing phenomenon playing important ecological roles in unicellular microorganisms. Here, we review the current progress and the existing knowledge gap regarding apoptosis-like death in *Microcystis*. Specifically, we focus first on the tools utilized to characterize the apoptosis-related biochemical and morphological features in *Microcystis*. We further outline various stressful stimuli that trigger the occurrence of apoptosis and discuss the potential mechanisms of apoptosis in *Microcystis*. We then propose a conceptual model to describe the functional coupling of apoptosis and MC in *Microcystis*. This model could be useful for understanding both roles of MC and apoptosis in this species. Lastly, we conclude the review by highlighting the current knowledge gap and considering the direction of future research. Overall, this review provides a recent update with respect to the knowledge of apoptosis in *Microcystis* and also offers a guide for future investigations of its ecology and survival strategies.

## 1. Introduction

*Microcystis* is a unicellular, planktonic, freshwater cyanobacterium with a global distribution [1]. It is also the most common bloom-forming cyanobacterium in eutrophic waters. *Microcystis* abundance is typically associated with warm water temperature, and *Microcystis* also overwinters as benthos in the temperate zone [2]. Under the present scenario of both the warming climate and the increasing eutrophication, the duration, frequency, and magnitude of *Microcystis* blooms are anticipated to increase as discussed elsewhere [3].

The potential expansion of *Microcystis* is of particular concern. This is mainly because it can form massive water blooms and deteriorate the functionality of ecosystems, moreover, some *Microcystis* strains are capable of producing the potent toxin known as microcystins (MCs) [4]. These compounds belong to a group of cyclic heptapeptides which act mainly on hepatocytes via inhibiting protein phosphatases 1 and 2A, eventually causing liver damage [5]. MCs have been also found to potentially adversely affect other animal organs such as the small intestine, colon, brain, kidney, lung, and heart as well as the reproductive system [6]. Additionally, MCs also have potential tumor-promoting activity [7]. These metabolites are not produced exclusively by *Microcystis*; other freshwater cyanobacteria e.g., *Anabaena*, *Planktothrix*, *Nostoc*, and *Oscillatoria* can produce MCs [4]. Recently, a *Raphidiopsis* strain (previously *Cylindrospermopsis raciborskii*) isolated in Greece has been identified as a potent MC producer [8]. To date, more than 200 congeners of MCs have been identified in cyanobacteria [9], MC-LR is the most common congener and receives the most attention [10]. As MCs threaten public health, the World Health Organization has set a guideline of 1 µg/L of MC-LR in drinking water [11]. Genetically, MCs are non-ribosomally biosynthesized through a thio-template mechanism involving multiple enzymes, e.g., peptide synthetases, polyketide synthases, putative transporters, and tailoring enzymes. The gene cluster of the MC biosynthesis pathway in *Microcystis* consists of ten genes (*mcyA–J*, approximately 55 kb in length) [12,13]. With the establishment of a genetic basis of MCs biosynthesis, various molecular approaches have been subsequently developed for detecting and quantifying the potential of MC-producing cyanobacteria [14,15]. While the biosynthesis, identification, detection, and toxicity of MCs have been well understood [16], the biological and ecological functions of these metabolites still remain a subject of debate [17]. Various functional hypotheses about their extracellular and intracellular role have been put forward [18,19,20], e.g., promoting iron acquisition and nutrient metabolism [21,22], defending from grazer attacks [23], protecting from oxidative stress [24], acting as quorum-sensing chemicals [25], involving info-chemical signaling [26], participating in light adaptation [27], acting as an allelopathic chemical [28], and promoting the formation of a *Microcystis* colony [29]. In the natural environment, MC-producing and non-MC-producing strains of *Microcystis* coexist [30], and increasing evidence suggests that, under particular conditions e.g., warmer temperature and oxidative stress, MC-producing *Microcystis* strains can exhibit a significant growth advantage over non-MC-producers [31]. This further implies that, with global climate warming, MC-producing *Microcystis* blooms would be more dominant than the non-MC-producing strains via a so-called positive feedback loop mechanism [32]. 

As such, controlling and managing *Microcystis* blooms has not only become crucially important, but also challenging. This is associated with the complicated biological traits of *Microcystis* blooms and the coupling of the death of *Microcystis* and the release of MCs. For example, chemical measures (hydrogen peroxide and copper sulfate etc.) can effectively kill *Microcystis* cells but simultaneously cause a significant release of toxins from the dying *Microcystis* cells [33,34]. MCs are naturally intracellular or cell-bound (they stay within intact cells, and are released following cell death or injury) [23]. Structurally, MCs are relatively stable and resistant to harsh physical conditions (e.g., high temperature); therefore, removing the extracellular fraction can be more challenging than removing their cell-bound form during the process of water treatment. In water treatment plants, the removal of MCs relies mainly on advanced treatment technologies including activated carbon absorption, which are usually not routine tools due to their relative cost. These measures for controlling *Microcystis* blooms thus augment, rather than cope with the existing problem of the water quality. Moreover, the MCs released into the environment can exert their extracellular and ecological function, which may be associated with the dominance of *Microcystis*. Additionally, the process of *Microcystis* death (especially under a massive bloom scenario) can also have a significant impact on the aquatic life and even global biogeochemical cycles. In such a context, understanding both the cellular death of *Microcystis* and the release of MCs is essential from the perspectives of public health and ecology. 

The mode of cell death has been investigated primarily in the multicellular metazoan; it typically consists of two primary types: necrosis (often called “accidental cell death”) and programmed cell death (PCD). The apoptosis that is often mentioned is a form of PCD [35]; the former is a passive, uncontrolled process driven abruptly by external extreme stimuli (e.g., heat and toxins) which cause the acute damage of the cellular membrane and rapid cellular death. By contrast, the latter is an active progress tightly controlled by a set of specialized molecular machinery which is activated by external or internal stimuli (e.g., oxidative stress and aging). Although PCD is a biological activity to counter the cellular proliferation, it plays an important role in maintaining homeostasis, development, and the functionality of multicellular organisms [36]. However, our knowledge of the cell death of unicellular phytoplankton (regardless of eukaryotic microalgae or cyanobacteria) remains relatively limited [37,38,39,40,41]. In the natural environment *Microcystis* must have evolved a series of ecological mechanisms to cope with adverse abiotic and biotic stress (e.g., ultraviolet irradiation) [42,43]. In the past two decades, studies have demonstrated the occurrence of apoptosis-like death in both toxic and nontoxic *Microcystis* strains under various environmental stresses [44,45,46,47,48,49,50,51,52,53,54] and the existence of the caspase homolog in *Microcystis* [55,56]. This raises a number of questions regarding the physiological and ecological relevance of apoptosis-like death in *Microcystis*.

This review aims to outline the current progress and the existing knowledge gap regarding apoptosis research in the freshwater bloom-forming cyanobacterium *Microcystis*. Specifically, we focus on (1) reviewing various approaches used to detect apoptosis-like death in *Microcystis*; (2) reviewing the reported factors (e.g., H_2_O_2_ and allelopathic chemicals) that can induce this process in *Microcystis,* and discussing the potential mechanisms behind it; (3) proposing a conceptual model with respect to the functional coupling of apoptosis-like death and MCs in *Microcystis;* and (4) outlining the existing knowledge gap and the future research direction in this field of study. 

## 2. Detection of Programmed Death in *Microcystis*

Programmed cell death (PCD) in a metazoan is a complex biological process that is mediated by various cellular signaling pathways, however, the classic PCD relies on a highly conserved family of proteolytic enzymes known as caspases (cysteine aspartyl-specific proteases), although one should note that caspase-independent pathways of PCD also exist [35]. Of note, the apoptosis termed in most literature almost exclusively refers to the caspase-dependent PCD. The proteolytic activity of caspases has been utilized as the most common hallmark of the apoptosis event [57]. In addition, the cells that undergo apoptotic death exhibit morphological and biochemical characteristics that are distinct from those of intact and necrotic cells. These distinct features include cell shrinkage, the condensation of nuclear material (chromatin), the activation of endonucleases, the fragmentation of DNA resulting from cleavage of the genome by endonucleases, and externalization of phosphatidylserine (PS) [57,58]. All these specific features have been exploited to develop the benchmarks to discriminate viable, apoptotic, and necrotic cells. So far, the most common technologies utilized to detect apoptosis include transmission electronic microscopy (TEM, for studying morphological change), gel electrophoresis (detecting DNA fragmentation), terminal deoxynucleotidyl transferase dUTP nick end labeling (TUNEL) assay (detecting DNA fragmentation), caspase detection assay, and Annexin V assay (detecting the externalization of phosphatidylserine) [58]. In a pioneering work that set out to study the cell death of *Microcystis*, Sigee et al. utilized multiple staining techniques (Evans Blue, Hoechst staining, and TUNEL) to study PCD-like death in the *Microcystis* field samples [44]; and Li and Song developed an optimal, colorimetric MTT assay to measure the viability of eukaryotic algae and cyanobacteria, specifically for *Microcystis aeruginosa* [59], and a fluorescein diacetate assay was later developed and discussed for measuring the metabolic activity of *Microcystis aeruginosa* [60]. All the above mentioned methods together constitute an important technique basis for studying apoptosis-like death in *Microcystis* and are further discussed in the following subsections. 

### 2.1. Transmission Electronic Microscopy (TEM) 

TEM is a powerful, high-resolution microscopic imaging technology for analyzing cellular ultrastructure at the nanometer scale. Thus, TEM can provide a detailed characterization of the morphological change in cell wall and membrane as well as cellular inner structural composition in the apoptosis event. Ding et al. using the TEM approach, demonstrated the remarkable structural difference between viable and H_2_O_2_-induced apoptotic *Microcystis* cells [48]; specifically, the occurrence of cell shrinkage (a typical indicator of apoptosis) was shown in H_2_O_2_-treated cells. Moreover, compared to living cells, the apoptotic fraction displayed a less dense stroma, deformation of the cellular membrane (wavy-folding outer cell membrane), and disintegration of thylakoids as well as cytoplasmic vacuolation. Yang et al. employed the TEM approach and also observed cell shrinkage in *Microcystis* cells undergoing apoptotic-like death induced by algicidal prodigiosin from the bacterium *Hahella* [45]. Interestingly, they further observed the budding and formation of apoptotic bodies at the late stage of apoptosis. Likewise, cell shrinkage and vacuolation have also been demonstrated in apoptotic cells of *Microcystis* induced by polyphenolic allelochemical pyrogallic acid [51], while the nucleoid disappeared with photosynthetic lamellae breaking [51]. Additionally, using TEM technology, He et al. showed the disintegration of thylakoids, loss of cytoplasm density, a fuzzy nuclear zone, cytoplasmic vacuolation, and disintegration of thylakoids in the apoptotic *Microcystis* cells induced by the allelopathic effect of the submerged macrophyte *Myriophyllum spicatum* in a co-culture system [52]. Overall, these TEM observations highlighted that cell shrinkage, vacuolation, and the disappearance of the nucleoid seemed to be conserved events during the development of apoptosis-like death in *Microcystis*. 

### 2.2. Gel Electrophoresis

Gel electrophoresis is a common tool for analyzing DNA fragmentation. During the apoptosis stage, the activities of endonucleases significantly increase, and DNA is cleaved into small pieces (fragmentation) [57]. In previous studies, DNA fragmentation was observed in the apoptotic cells of *Microcystis*. For example, using gel electrophoresis, He et al. demonstrated a progressive amplification of the degree of DNA fragmentation in apoptotic cells of *M. aeruginosa* FACHB905 induced by the allelopathic effect of submerged macrophyte *Myriophyllum spicatum* [52]. Ding et al. also observed increasing DNA fragmentation in apoptotic *Microcystis* when exposed to increasing H_2_O_2_ concentration (from 250 µM to 325 µM) or for a longer stress time (from 24 to 48 h) at a concentration of 250 µM or 325 µM of H_2_O_2_) [48]. Lu et al. observed that accompanying the apoptosis event of *M. aeruginosa* induced by polyphenolic allelochemical pyrogallic acid, the level of genomic DNA fragmentation of *Microcystis* increased over the concentration gradient of this acid [51]. However, Yang et al. recently reported that DNA fragmentation did not necessarily accompany the apoptosis event in *Microcystis* induced by bacterial prodigiosin from *Hahella,* although other apoptotic-specific morphological and biochemical hallmarks were simultaneously presented. This phenomenon may be associated with the activation of the DNA excision repair system induced by prodigiosin [45]. Of note, unlike the DNA laddering pattern in plant and animal apoptotic cells, DNA fragmentation in *Microcystis* apoptotic-like cells was characterized by smearing in the gel electrophoresis [48,51,52]. This is mainly because cyanobacteria lack the chromosomal histone that otherwise exists in eukaryotic plant and animal cells as discussed elsewhere [51]. Based on these observations, fragmentation of DNA was still a common feature for the apoptosis event of *Microcystis,* although it was not observed in the apoptosis event of *Microcystis* induced by bacterial pigment prodigiosin from *Hahella* [45]. 

### 2.3. Terminal Deoxynucleotidyl Transferase dUTP Nick End Labeling (TUNEL) Assay 

TUNEL assay is another common approach used to detect the fragmentation of DNA during the apoptosis event. This method relies on an enzyme, terminal deoxynucleotidyl transferase, that catalyzes the addition of dUTP nucleotides to the 3’-hydroxyl (3’-OH) end of the cleaved (or fragmented) DNA [58]. So far, various types of TUNEL assays have been developed; in principal, these methods generally utilize the labeling of dUTPs that subsequently yield fluorescence or colorimetric signals, indicating the occurrence of DNA cleavage and fragmentation. Compared to gel electrophoresis, the TUNEL assay has outstanding advantages in qualifying the presence of DNA fragmentation at the single cell level.

In a pioneering study, Sigee et al. utilized the TUNEL assay to detect the DNA fragmentation in *Microcystis* colonies collected during the late summer bloom. This suggested that PCD was involved in the senescence of *Microcystis* blooms [44]. Using the fluorescent TUNEL microscopic assay, Bouchard and Purdie also demonstrated the presence of DNA degradation in the *M. aeruginosa* CCAP 1450/16 when exposed to darkness and H_2_O_2_-supplemented conditions; the percentage of TUNEL-positive *Microcystis* cell increased significantly, reaching ∼30% and ∼50%, respectively, compared to the control group [61]. Additionally, when exposed to H_2_O_2_ (250–325 µM for 24–48 h) and UV-B stresses, *Microcystis* exhibited a clear signal indicating an increasing degree of DNA fragmentation, which was detected by the fluorescent TUNEL microscopic assay [46,48]. These consistent observations from TUNEL fluorescence microscopic assay further showed that DNA fragmentation is conservatively present in the apoptosis-like death of *Microcystis*. 

### 2.4. Hoechst Staining Assay

The Hoechst staining assay is also a useful tool for observing the compact chromatin of the apoptotic cells. Hoechst stains are a group of cell-permeable nucleic acid dyes that produce a bright blue fluorescence when bounded to DNA. Hoechst 33342 is the most commonly applied stain for this purpose. In comparison to live cells, apoptotic cells typically exhibit increased permeability of the cell membrane and the condensation of DNA (the latter does not occur in necrotic cells), therefore, apoptotic, necrotic, and intact cells can be distinguished by Hoechst staining assay; the apoptotic cells typically have a brighter intensity of Hoechst fluorescence and simultaneously display an uneven distribution of Hoechst fluorescence under fluorescent microscopy [62]. Sigee et al. previously utilized Hoechst 33342 staining combined with TUNEL assay to analyze *Microcystis* field samples and found a considerable variation between colonies, with highly condensed nucleoid chromatin in the center of cells (suggestive of PCD) in some colonies and with more diffuse, less well-stained chromatin in other cells. Such a clear discrepancy exactly matched the difference reflected in TUNEL staining analysis: The condensation of chromatin (observed in Hoechst staining) occurred in the TUNEL-positive *Microcystis* cells whereas the diffuse Hoechst staining occurred in the TUNEL-negative *Microcystis* cells. Such a strong correlation of the results between Hoechst staining and TUNEL assay further supported the occurrence of PCD in *Microcystis* during late summer blooms [44]. Recently, Ding et al. also utilized Hoechst 33342 staining and observed significant chromatin condensation in these H_2_O_2_-treated *Microcystis* that underwent the process of apoptosis-like death [48]. These consistent observations in laboratory and field samples of *Microcystis* implied that Hoechst staining assay can also be a useful tool to study apoptosis in *Microcystis*. 

### 2.5. Caspase Assay

Caspase activation is a highly conserved feature of the early apoptosis stage in metazoa. So far, various caspase activity assays have been developed to determine the occurrence of apoptosis. Generally, these assays utilized the fluorogenic peptide substrates that subsequently yield the fluorescence signal, indicating the activity of caspase. Caspase assay is also the most common approach used to diagnose the occurrence of apoptosis-like death in *Microcystis*. For the first time, Ross et al. detected the elicited activity of caspase in H_2_O_2_-treated (10 and 100 µM) cells of *Microcystis*, suggesting the possibility of an H_2_O_2_-inducible apoptosis pathway [53]. He et al. used a commercial Caspase-3 Assay Kit and observed increasing caspase activity in *M. aeruginosa* when exposed to an increasing mass of the submerged macrophyte *Myriophyllum spicatum* (0.5, 1.0, 3.0, and 6.0 g/L, fresh weight), or when exposed for a longer period of time (up to 7 days) to a *M*. *spicatum* biomass of 6.0 g fresh weight (FW) L^−1^ [52]. Likewise, using a Caspase-3 assay, Lu et al. observed a notable increase of caspase-3(-like) activity in *M. aeruginosa* treated with 14 mg/L of polyphenolic allelochemical pyrogallic acid (PA), whereas a mild elevation of activity was observed with 2 and 7 mg/L of PA. These results indicated the involvement of a caspase-like enzyme in PA-induced growth inhibition of *M. aeruginosa* [51]. Ding et al. used the CaspGlow Fluorescein Active Caspase-3 kit and showed that the caspase-3-like activity of *M. aeruginosa* gradually increased after exposure to 250 and 325 µM H_2_O_2_ from 2 h to 8 h, compared to controls [48]. Likewise, using the CaspGlow Fluorescein Active Caspase-3 kit, Zhou et al. recently demonstrated that the exposure at 0.4 mM and above H_2_O_2_ led to the activation of caspase-3 in *M. aeruginosa* cells on the first day, indicating apoptosis-like death in the early stage of cell death [47]. Bouchard and Purdie utilized the Caspase-3 Assay Kit and found a significant increase in the activity of caspase-3 in *M. aeruginosa* under dark and H_2_O_2_-supplemented conditions at 96 h, compared to the controls [61]. Recently, Ross et al. utilized the Caspase-3 Assay Kit to demonstrate that the metacaspase-like activity in *M. aeruginosa* significantly increased in response to elevated salinity and corresponded to enhanced production of reactive oxygen species [63]. Using the Caspase-3 Assay Kit, Chen et al. observed an increasing trend of caspase-3-like levels in *M. aeruginosa* during exposure to 0.6 mM of vitamin C for 2 days [64]. Therefore, the increased activity of caspase-like appeared to be a conserved hallmark of apoptosis-like death in *Microcystis*. 

### 2.6. Annexin V Staining Assay

Annexin V staining assay is also a common approach that is particularly effective at identifying the early apoptosis event [65]. In principal, this method relies on a specific biological trait of a protein, Annexin V that preferentially binds phospholipid species e.g., phosphatidylserine (PS) in a Ca^2+^-dependent manner [65]. In viable cells, PS is located in the inner layer of cellular plasma membrane, therefore it cannot be stained by (bound with) Annexin V; however, in the early stage of apoptosis, PC can be translocated to the outer layer of cellular plasma membrane while the entire cell membrane itself remains intact, this allows Annexin V to bind with the PS exposed on the surface of external membrane. Of note, the exposure of PS is not unique to apoptosis, it also occurs in necrotic cells [66]. Therefore, in practice, Annexin V labelled with a fluorescent tag (e.g., FITC) is combined with a cell membrane-impermeable, nucleic acid dye (e.g., propidium iodide, PI). Subsequently, the cells dually stained by Annexin V-FITC/PI are applied to flow cytometry to differentiate viable cells (Annexin-FITC^-^/PI^-^) and cells undergoing early apoptosis (Annexin-FITC^+^/PI^−^), late apoptosis or necrosis (Annexin-FITC^+^/PI^+^), artifact or necrosis (Annexin-FITC^-^/PI^+^), as described elsewhere [65]. 

Wu et al. utilized the Cell Meter^TM^ Phosphatidylserine Apoptosis Assay Kit (Green Fluorescence) and flow cytometry, and detected an increasing proportion of phosphatidylserine exposure-occurring cells in *M. aeruginosa* culture as the treatment concentration of the herbicide glyphosate increased (0, 1, 2, 5, and 10 mg/L), indicating the occurrence of apoptosis in the studied cells [50]. Yang et al. utilized the dual staining assay of Annexin V- fluorescein and PI to analyze *M. aeruginosa* FACHB1752 treated with algicidal prodigiosin and found that the proportion of cells with PI positive staining or Annexin V-Fluos positive staining increased with the treatment time of prodigiosin, indicating that this compound caused both the externalization of phosphatidylserine and the loss of membrane integrity. This suggested that the apoptosis-like and necrosis-like events co-occurred in prodigiosin-stressed cells [45]. Likewise, using Apopxin™Green assay, Du et al. observed that the apoptosis rates in both *M. aeruginosa* and *M. viridis* increased over the applied concentration of the herbicide fenoxaprop-p-ethyl (0, 1, 2, 5, and 10 mg/L) [49]. Recently, Ye et al. utilized a Cell Meter™ Phosphatidylserine Apoptosis Assay Kit and observed an increased proportion of apoptotic cells in glyphosate-treated *M. viridis* FACHB979 as the concentration of herbicide increased (from 0.2 to 10 mg/L) [67]. However, Lu et al. who utilized the dual staining assay of Annexin V-FITC/PI to analyze *M. aeruginosa* DIANCHI905 treated by pyrogallic acid, did not detect any distinct externalization of phosphatidylserine, although other apoptosis-specific features (the activation of caspase-3, the disappearance of the nucleoid, and the fragmentation of DNA) were simultaneously detected [51]. This may be associated with the potential discrepancy in the timing of phosphatidylserine exposure between unicellular phytoplankton and multicellular organisms [68]. Thus, based on the inconsistent observation of phosphatidylserine exposure in the above mentioned studies, whether the Annexin V binding is a reliable biomarker of apoptosis in the *Microcystis* remains to be further investigated.

Collectively, all these data derived from various characterizations highlighted that the morphological and biochemical features (cell shrinkage, vacuolation, DNA fragmentation, nucleoid condensation, and the activation of caspase), which are the typical hallmark of apoptosis in the multicellular metazoa, also occurred in company with the cellular death of *Microcystis* regardless of the types of abiotic stress and *Microcystis*. Additionally, all these observations constitute important evidence to support the notion that caspase-like-involved PCD occurred in the freshwater bloom-forming cyanobacterium *Microcystis*. It is also recommended that multiple tools (at least three to four different characterizations) should be used simultaneously to better describe the morphological and biochemical features (cell shrinkage, DNA fragmentation, nucleoid condensation, and the activated caspase) of PCD in cyanobacteria. 

## 3. The Mechanisms of Programmed Death in *Microcystis*

So far, a total of 10 major types of abiotic stress factors have been reported to be capable of triggering the occurrence of apoptosis-like events in *Microcystis*: exogenous oxidants (e.g., H_2_O_2_), excessive salt, darkness, high concentration of vitamin C, aldehydes (e.g., cinnamaldehyde), herbicides (fenoxaprop-p-ethyl, glyphosate, and methyl viologen), allelochemicals (pyrogallic acid and phenolic compounds), bacterial pigment e.g., prodigiosin, ultra-violet irradiation, and high pH (Table 1). Consistently, these abiotic stress factors are either a direct oxidant or can trigger the production of internal reactive oxygen species (ROS) in *Microcystis*. Specifically, Ross et al. detected the occurrence of PCD in *Microcystis* because of oxidative stress either directly derived from treatment with H_2_O_2_ (10 µM) or generated from the treatment of salinity, physical injury, herbicide (methyl viologen), and UV irradiation [53]. Sigee et al. suggested that the occurrence of PCD in *Microcystis* colonies in late summer bloom may be attributed to the generation of ROS derived from a high epilimnion pH value (pH = 9.2) and depletion of dissolved CO_2_ in a eutrophic lake [44]. Hu et al. detected increased H_2_O_2_ generation soon after the treatment of toxic *M*. *aeruginosa* FACHB905 with cinnamaldehyde (0.6 mM), concomitant with the occurrence of PCD. Importantly, simultaneous treatment with antioxidants (glutathione and vitamin C) was shown to ameliorate cinnamaldehyde-induced cell lysis. This additionally suggested that the cinnamaldehyde-triggered PCD in *M*. *aeruginosa* FACHB905 was associated with the formation of a cinnamaldehyde-induced ROS burst [54]. Ding et al. suggested that the occurrence of apoptosis in *Microcystis* can be associated with the generation of the oxidative stress induced by UVB irradiation [46]. In turn, Wu et al. found that the allochemical pyrogallic acid (PA) from the aquatic vascular plant *Myriophyllum spicatum* and its autoxidized products were involved in a futile redox cycle which mediates the production of ROS (O^2.^, H_2_O_2_, and OH^˙^) in *Microcystis*, and the PA-induced ROS acted as a mediator and led to the inactivation of caspase-like and the subsequent initiation of PCD in *Microcystis* [51,52,69]. Wu et al. also showed that increased oxidative stress accompanied the occurrence of apoptosis in *Microcystis aeruginosa* when treated by the herbicide glyphosate (1 and 2 mg/L), and as speculated, the glyphosate caused oxidative stress damage in *Microcystis* that further triggered the occurrence of apoptosis in treated cells [50]. Yang et al. simultaneously detected the generation of ROS and the occurrence of PCD in *M. aeruginosa* when treated by algicidal prodigiosin (PG) from *Hahella* sp. KA22. As proposed, PG-induced ROS formation was involved in the occurrence of the PCD [45]. Interestingly, Chen et al. demonstrated that vitamin C (0.6 mM) triggered the occurrence of apoptosis in toxic *M. aeruginosa* FACHB905 via the Fenton reaction in which, vitamin C enhanced iron absorption leads to high ferrous ion levels that subsequently increase the production of ROS [64].

These findings consistently point to a link between the occurrence of apoptosis and the generation of ROS in stressed *Microcystis* cells. Indeed, (ROS are a key inducer of apoptosis extensively demonstrated in animal, higher plant, and yeast as well as unicellular eukaryotic and prokaryotic algae [38,70,71]. As also shown, pretreatment with antioxidants can protect the cells against the occurrence of apoptosis, which was also shown in reductant-treated *Microcystis* [54]. Interestingly, at higher concentrations antioxidative compounds such as vitamin C can also induce PCD [64]. In a general perspective, the observed apoptosis-like death in *Microcystis* under various stress stimuli (e.g., herbicide, UV irradiation, and high pH) resulted from either direct oxidant treatment (H_2_O_2_) or indirect generation of intracellular oxidative stress by stimuli. At present, the detailed apoptotic pathway in *Microcystis* remains unknown and yet to be explored. However, a speculation can be appropriately made from the existing clues in *Microcystis* and the established mechanisms of PCD in eukaryotes and other well-studied bacteria (e.g., *Escherichia coli*) [37,72,73,74,75,76], as well as the marine bloom-forming cyanobacterium *Trichodesmium* [77]. 

In the animal model, apoptosis occurs via two major signaling pathways [78]: the intrinsic and extrinsic one (one can affect the other).The former is also known as the mitochondrial pathway, and is initiated by intracellular stress events (e.g., oxidative stress and DNA damage) that can be caused directly or indirectly by a broad array of exogenous and endogenous cytotoxic stimuli (e.g., hydrogen peroxide and ultra-violet irradiation), such intracellular stress, targets the mitochondria and results in permeabilization of the mitochondrial outer membrane (MOMP) and the subsequent release of death-promoting proteins (e.g., cytochrome C). This further activates the caspase cascade, causing a series of cellular deconstruction activities (degradation of protein, fragmentation of DNA, and externalization of phosphatidylserine as well as morphological change) and eventual cellular death [78]. The extrinsic pathway is independent of mitochondria, instead, it is initiated by the extracellular ligand-receptor binding on the cell-surface, which directly activates the caspase cascade and eventually causes cellular death [78].

In prokaryotic bacteria, various genetic regulation modes (e.g., toxin–antitoxin module, and *recA*/*lexA*-mediated apoptotic-like death system, holing-endolysin system, thymine-less death) have been described for understanding the mechanisms of PCD [72,74,75,76]. The toxin-antitoxin (TA) system (e.g., MazEF TA system) is the most common and has been demonstrated in detail in *Escherichia coli*. The toxin (MazF) in the TA system inhibits cellular growth, whereas the labile antitoxin (e.g., MazE) counteracts the toxin (MazF). When exogenous or endogenous cytotoxic factors (e.g., oxidative stress, antibiotics, and viruses) cause significant inhibition of antitoxins and the subsequent failure of the antitoxin in counteracting the toxin, this will eventually result in cellular death [74]. Of note, the role of TA is still debated; it has been suggested that the TA system is associated only with the induction of bacterial static status, rather than with cell killing as expected [79]. Hu et al. surmised the existence of a module similar to the bacterial TA system that triggered the occurrence of PCD in the cyanobacteria [54], however, the apoptosis-specific features (e.g., the activation of caspase) have seldom been documented for the TA system-mediated bacterial PCD. In addition, the effect of DNA damage on the fragmentation of DNA was found to be inhibited by the TA system MazEF-mediated cell death pathway in *E. coli* [80]. Recently, Klemenčič and Dolinar have identified two pairs of TA system genetic loci (*relBE* and *vapBC*) located downstream of the orthocaspase MaOC1 gene (encoding an orthocaspase) in the genome of *M. aeruginosa* PCC7806. However, their genomic data-based analysis did not eventually characterize any clear role of the TA system in the PCD of *Microcystis* [81]. Logically, all these limited hints together negate a significant role of the TA system in regulating the caspase-like-involved PCD in *Microcystis*, but one cannot completely rule out the possibility that the TA system has an indirect association with the PCD of *Microcystis*. 

By contrast, *recA*/*lexA*-mediated death in bacteria accompanies a series of biochemical and morphological changes (e.g., externalization of phosphatidylserine, the condensation of chromosome, and fragmentation of DNA as well as the activation of caspase) [72,82], which are similar to the hallmarks of caspase-dependent apoptosis in metazoa. Therefore, *recA*/*lexA*-mediated cellular death is known as the apoptosis-like death (ALD). LexA acts as the repressor of the SOS response genes and is thus the general repressor of the SOS response in a normally healthy cell, however, under a stressful condition, RecA is activated to stimulate the cleavage of the LexA repressor and activates the SOS regulatory response [83]. Moreover, RecA binds with the substrates of metazoan caspases clearly implying a caspase-acting role of RecA [82]. Since the morphological and biochemical features of apoptosis in *Microcystis* resemble those observed in *recA/lexA*-mediated bacterial death, one can ask whether the *recA/lexA*-mediated pathway is involved in apoptosis in *Microcystis*. Currently, no experiments have been performed to directly answer this question. However, interestingly, bioinformatics analysis revealed that *lexA* indeed exists in the genomes of selected (but not all) cyanobacteria species including *Microcystis*. However, cyanobacterial LexA does not always regulate the SOS response and it appears that it has otherwise adopted another unique physiological role [84]. For example, Honda et al. recently showed that LexA in *M. aeruginosa* NIES843 may function as a transcriptional activator of genes related to cell reproduction [85]. Likewise, these limited clues together indirectly negate a major role of *recA* in regulating the PCD observed in *Microcystis* despite the similarity of phenotypical features between apoptosis-like death in *Microcystis* and the *lexA*-mediated apoptosis-like death in bacteria. 

The third group of intriguing clues comes from extensive studies of metazoan caspases and their homologs in plants, yeasts, eukaryotic algae, and bacteria, including cyanobacteria, especially *Microcystis* [56,57,86,87,88]. In the metazoan, caspases are the central executioners of PCD; structurally, they have a highly conserved active-site cysteine and specifically cleave the substrate after the aspartate residue (Asp). The apoptotic morphological features result directly from the activated caspase cascade [57]. However, non-metazoan organisms do not have true caspases, but some have various caspase-homologous proteins (metacaspases). In spite of the structural similarity with the metazoan caspase, the metacaspase exhibits Arg or Lys specificity, which is different from the Asp specificity of the metazoan caspase. Speculatively, the metazoan caspases and their homologs have a common ancient ancestor and the metazoan caspase has evolved from α-proteobacteria and cyanobacteria [88]. In function, metacaspase was initially hypothesized to be the counterpart of metazoan caspase, and to initiate and execute the PCD in non-metazoa as does the caspase in metazoa. However, extensive laboratory and field studies brought in an intricate scenario that metacaspases have multifaceted functions involved in either PCD-associated or non-PCD-associated activity [86]. Evidence to support the PCD-associated roles are derived from a series of experiments in the higher plant (*Arabidopsis*) [89], yeast (*Saccharomyces cerevisiae*) [90], and eukaryotic algae (marine diatom *Phaeodactylum tricornutum*) [91], as well as the bacterium *Xanthomonas* (γ-proteobacteria) [92]. As shown, inactivation or inhibition of the metacaspase in these organisms can significantly reduce or completely abrogate the occurrence of the apoptosis-like events. However, genetic or chemical disruption experiments also demonstrated that metacaspase plays an important role in non-PCD-associated biological progress. For example, yeast metacaspase Yca1p accelerates G1/S transition and slows G2/M transition [93]. 

Of note, currently, research into the function of metacaspase in cyanobacteria remains in its infancy, and existing knowledge is based mainly on bioinformatics analysis [87,94,95,96] and the biochemical and transcriptional exploration of limited laboratory and field samples [97], mainly in the freshwater bloom-forming cyanobacterium *M. aeruginosa* [56], and marine bloom-forming, filamentous cyanobacterium *Trichodesmium* [77,98]. The metacaspase gene is almost ubiquitous in the cyanobacteria except *Prochlorococcus* and some strains of *Synechococcus*; and it is relatively abundant in the filamentous diazotrophic cyanobacteria (e.g., *Trichodesmium*, *Nostoc*, and *Anabaena*). Its presence and quantity in the cyanobacterial genome seem to be associated with genome size, morphological and physiological complexity, and the ecological habitat of cyanobacteria [87,94,95,96]. In the genome of *M. aeruginosa* PCC7806, six putative metacaspase genes (*MaOC*1–6) have been identified; and the MaOC1 was recently expressed in *E. coli* [55]. Characterization work revealed that MaOC1 is a highly active proteolytic enzyme with a preference for arginine at the P1 position and an optimal pH at 7.5, which are in accordance with those of previously reported metacaspases [55]. Due to the observed structural difference to other metacaspases in plants and yeast, MaOC1 together with other bacterial metacaspases it was proposed that they be termed as orthocaspases. Given the observed distinct substrate specificity between caspase and bacterial orthocaspase, the previously detected caspase-like activity in the bacteria including cyanobacteium *Microcystis* is most likely attributed to a yet unidentified protease, other than the bacterial orthocaspase [56]. Additionally, overexpression and accumulation of MaOC1 did not cause cellular death in the expression host system *E. coli*, which was otherwise observed for expression of toxins of TA systems in *E. coli*. This implied that orthocaspase MaOC1 might be not the real executors of PCD [81]. However, this needs to be further clarified in *Microcystis*. 

The non-PCD-associated roles of cyanobacterial metacaspase were also implied in a recent microbial community-wide metacaspase survey [97]. This study performed metagenomic and metatranscriptomic analysis on samples collected in the brackish Baltic Sea where massive cyanobacterial blooms periodically occur; the metacaspase genes of Baltic Sea filamentous cyanobacteria were found to be constitutively expressed throughout the season, rather than limited to a bloom period. In addition to the potential PCD-associated function, cyanobacterial metacaspases were speculated to have potential roles in house-keeping functions and stress adaptation (e.g., in sulfur metabolism under oxidative stress, and in cellular differentiation under nutrient stress). However, a pro-cell death-associated role of cyanobacterial metacaspase was implied in a marine bloom-forming cyanobacterium *Trichodesmium*: Up to 12 metacaspase genes have been identified in this cyanobacterium [94]. 

A parallel elevation of the activity of caspase and the expression of metacaspase genes was observed in a marine cyanobacterium population that underwent apoptosis-like death [99]. In particular, the metacaspase TeMC of *Trichodesmium* was recently studied where it was found that the activity of metacaspase TeMC was significantly positively correlated with that of the caspase-like in the laboratory and field samples of *Trichodesmium* that underwent the apoptosis-like death [98]. This biochemical and physiological evidence directly linked the expression and activity of metacaspase with the occurrence of an apoptosis-like event in cyanobacterium, and this is similar to the PCD scenario in the marine diatom *Phaeodactylum tricornutum* [91] in which the PCD-associated role of metacaspase has been demonstrated by the overexpression and knockout experiment. Therefore, like the metacaspase in eukaryotic algae, cyanobacterial metacaspase might also play an important role in regulating PCD. Previous study has implied that caspase-like proteases may exist downstream of the metacaspase in the regulation pathway, and the activity of downstream caspase-like protease can be affected by the upstream caspase-like proteases [86,90,100]. This well explains the tight correlation between metacaspase and caspase-like activities observed in *Trichodesmium* [98]. However, one cannot rule out a possibility that the tight correlation between metacaspase and caspase activities might be associated with the activity increase of an entire set of proteolytic enzymes triggered by stimuli [87]. Overall, the cyanobacterial metacaspase could also have multifaceted roles involved in either PCD or adaptive response to an adverse environment. These abovementioned findings pave an important avenue to further study the role of the metacaspase in *Microcystis* and the mechanisms of the PCD in these cyanobacteria. 

## 4. The Functional Coupling between Apoptosis and Microcystin in *Microcystis*

So far, no specific studies have been performed to directly explore the physiological and ecological relevance of PCD in *Microcystis*. However, various functions of PCD in the unicellular metazoan and in unicellular bacteria and phytoplankton have been extensively discussed or reviewed elsewhere [36,38,39,72,73,74,75,76,101]. In the metazoan, PCD is an essential biological process that removes cells with molecular errors for maintaining cellular homeostasis and further development of the organism as an entity. In other words, PCD is certainly not beneficial for the individual cell undergoing this process, but eventually beneficial for the metazoan as an entity. However, for unicellular bacteria or phytoplankton, the counterproductive behavior of PCD does not offer any benefit to an individual cell as it halts its ability to proliferate. To understand the relevance of PCD in unicellular microorganisms, one must first accept two concepts: (i) the concept of a microbial clonal population or community: A microbial clonal population is an entity composed of multiple individual cells, which are inter-connected or social; and (ii) the concept of kin selection that argues that there exists an evolutionary strategy to favor the survival of genes which are present in an organism’s relatives [102]. As such, PCD is not beneficial for an individual cell; however, it eventually confers an advantage to the remaining clonal cells to maintain the microbial population as an entity under certain conditions. This scenario resembles that in multicellular metazoa. In other words, such bacteria PCD is altruistic and beneficial for the survival of genes which are present in other cells carrying it, and by which process it could be conserved through evolution [101]. 

Further, various advantages conferred on the bacterial population have been discussed previously and mainly involve the following five aspects [72,101,103]: (i) providing nutrients: PCD eliminates damaged cells and reduces the competition of the remaining clonal cells for the nutrients, thus eventually improving the fitness of an entire clonal population [103]; (ii) maintaining gene conservation: Some cells that harbor deleterious mutation might be removed by PCD, therefore, this ensures gene conservation for normal population proliferation [74]; (iii) cell–cell communication: The cells undergoing PCD release info-chemicals that might notify their neighbor clonal cells to adapt in a timely manner to changing stimuli [101]; (iv) defense from viral infection: PCD removes the virus-infected cells, thus indirectly mitigating the virus spread within the bacterial population [74]; and (v) improving the strength of bacterial biofilm: Genomic DNA released from the dying cells can act as an adherence molecule to strengthen the extracellular matrix or consolidate the bacterial biofilm [72]. *Microcystis* grows in the colonial form resembling the bacterial biofilm. Speculatively, these advantages also hold true for *Microcystis*.

**Figure 1:**
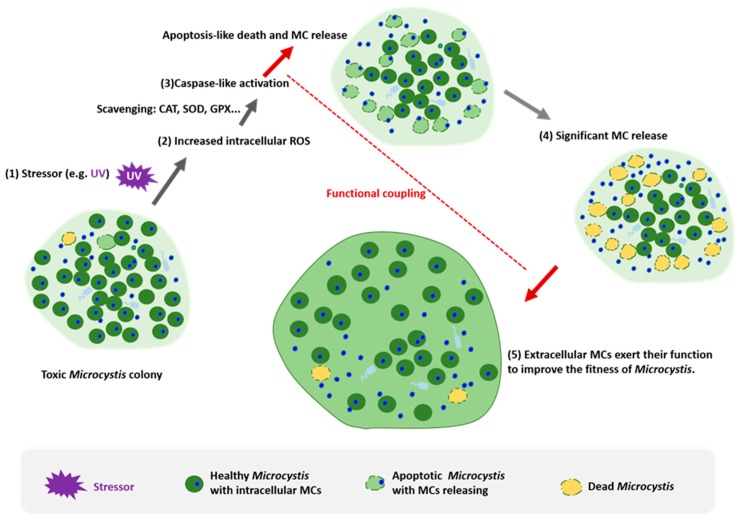
The hypothetical conceptual model coupling programmed cell death (PCD) and the role of microcystins (MCs) in *Microcystis*. (1) The extracellular stressor (e.g., ultraviolet radiation) acts on the cell. (2) Intracellular oxidative stress increases; the intracellular reactive oxygen species (ROS) content exceeds the antioxidative capacity of the cell (mediated mostly by an enzymatic system involving a superoxide dismutase (SOD), catalase (CAT), and glutathione peroxidase (GPX)) and causes molecular damage. (3) The damage further activates the caspase-like activity, and apoptosis-like death is initiated. Simultaneously, intracellular MCs begin to be released into the extracellular environment. (4) The extracellular MCs have been significantly released from dead *Microcystis* cells. (5) They act on the remaining *Microcystis* cells, and exert extracellular roles, for example, as previously demonstrated by Gan et al. [29], extracellular MCs can increase the production of extracellular polysaccharides (EPS) that are involved in colony formation. Eventually, the colonial form improves the survival of the remaining cells under stressful conditions.

Of note, the occurrence of the PCD in *Microcystis* essentially entails the significant release of MC into environment. MC is a typically intracellular metabolite but its extracellular multifunctional traits have been gradually recognized [18,19], for example in the aspects of nutrient uptake [21,22] and cell–cell communication [23,26]. Particularly, MC acts as a potent mediator in promoting the formation of a *Microcystis* colony [29]. The colonial trait is a crucially important morphological trait of *Microcystis* that contributes to the ecological dominance of *Microcystis*; one can thus envision a reasonable scenario: The occurrence of PCD in *Microcystis* under certain conditions causes the significant release of intracellular MC, subsequently, the MC exerts its extracellular functions that can be beneficial to the *Microcystis* population and eventually contribute to its ecological success, as illustrated in Figure 1. Since there exists an intrinsic co-occurrence of PCD and the release of MC in *Microcystis*, we further hypothesize here that there also exists an inherent functional coupling between the release of MC and PCD in *Microcystis* (Figure 1). Our hypothesis is based on multiple independent physiological and genetic evidence: (i) both (extracellular and intracellular) MC and PCD confer a relative physiological advantage to *Microcystis*, as discussed above; (ii) the occurrence of PCD naturally accompanies the release of MC as demonstrated elsewhere [49,53]; (iii) both PCD and MC production have an ancient evolutionary root; in evolution, MC production predated the metazoan lineage [104], and the core protease (caspases) of metazoan apoptosis evolved from ancient bacterial ancestors, and the metacaspases originated early in the evolution of life (∼3 billion years ago) [94], when cyanobacteria appeared; (iv) there exists a strong association between the induced apoptosis event and upregulated biosynthesis of MC in the remaining *Microcystis* cells. Kaplan and colleagues previously demonstrated that when *Microcystis* became aged or underwent the lysis triggered by the apoptosis inducer (H_2_O_2_) and allelochemicals, the MC synthetase McyB in the remaining surviving *Microcystis* cells massively accumulated [26]; (v) MC-producing and non-MC-producing *Microcystis* exhibited distinct modes of apoptosis-like death when exposed to harmful UV-B irradiation [46]; and (vi) last but not the least, there is a genetic connection between MC production and the PCD-associated metacaspase in cyanobacteria. Asplund-Samuelsson et al. recently reported the co-expression of the metacaspases and the synthesis enzymes of nodularin (the structural cousin of MC) in *Nodularia spumigena* [97]. In addition, non-MC-producing strains of *Microcystis* may potentially benefit from this strategy without the production of MC since the non-MC-producing strains often co-occur with the MC-producing one as actually observed in the field. Our present hypothesis will help to more comprehensively understand the relevance of the MC and the PCD in cyanobacteria, the life cycle of *Microcystis* as well as the ecological mechanisms of the formation of massive *Microcystis* blooms. One should note that MC can also be released to the environment via typical necrosis, although this process is not controlled intracellularly. The PCD mechanism allows the function of the released MC to be tightly controlled, which is likely to be advantageous compared to uncontrolled cell lysis. 

Due to the intrinsic coupling of the cell death of *Microcystis* and the release of MC, careful attention should be paid to the potential risk of increased MC issue when applying chemical and biological measures (e.g., H_2_O_2_ treatment and potential biological treatment) to control *Microcystis* blooms. As such, preventing the occurrence of massive *Microcystis* blooms should be more important than controlling and managing the existing *Microcystis* blooms in the long run. 

## 5. Future Research Prospects

So far, the apoptosis-like event has been consistently demonstrated in *Microcystis*, via the use of different physical, biochemical, and genetic approaches, and the metacaspase in *Microcystis* that is speculatively associated with PCD has been identified and characterized. These achievements in *Microcystis*, together with the previous findings in other cyanobacteria and bacteria, have constituted an important basis for directing further PCD study in *Microcystis*. Future work should focus on the following aspects: (i) to further study the scenario of PCD in the natural *Microcystis* samples in lakes; this could involve exploring various biotic and abiotic factors that can significantly trigger the occurrence of PCD in *Microcystis,* Particularly, it is of interest to study whether cyanophage can induce the occurrence of PCD in *Microcystis* since cyanophage serves as a potent biotic factor that kills *Microcystis* [105,106]; (ii) to dissect the molecular mechanisms by which PCD is initiated and executed in *Microcystis,* and to further explore the role of metacaspase in *Microcystis*; and (iii) to examine our present hypothesis of the functional coupling between MC and PCD in *Microcystis*. In addition, it would be also worth studying how the present model could fit into the survival of filamentous MC-producing cyanobacteria e.g., *Planktothrix agardhii*, which are not known to form colonies. However, under some stress stimuli, *P. agardhii* can increase in width, which might be involved in an adaptive defense mechanism [107]. These future works will certainly help to better understand the life cycle of *Microcystis* and other cyanobacteria, and to better guide the prevention and control of cyanobacterial blooms, as a step towards improving public health.

## Figures and Tables

**Table 1:** Summary of the occurrence of apoptosis-like death in *Microcystis* under various stimuli. Programmed cell death (PCD), terminal deoxynucleotidyl transferase dUTP nick end labeling (TUNEL), transmission electronic microscopy (TEM).

*Microcystis* Strain	Stimulus	Approaches for Analyzing PCD	ROS * Production	Reference
***M. aeruginosa***	H_2_O_2_ (100 µM)	Caspase-3 Assay	Yes	[53]
**Field *Microcystis* samples**	Natural environmental condition (e.g., pH > 9.2 and depletion of CO_2_)	Evans blue staining, Hoechst 33342 staining, and TUNEL assay	Yes	[44]
***M. aeruginosa* FACHB905**	Cinnamaldehyde (0.15–1.5 mM)	No available	Yes	[54]
***M. aeruginosa* CCAP** **1450 ⁄ 16**	Dark and oxidative stress (0.5 mM)	Caspase 3-like assay and TUNEL assay	Yes	[61]
***M. aeruginosa* FACHB905**	H_2_O_2_ (150–325 µM)	TEM, Hoechst 33342 staining assay, TUNEL assay, gel electrophoresis, and Caspase-3 assay	Yes	[48]
***M. aeruginosa* PCC7005, PCC7806, and FACHB905**	UV-B irradiation (0.5 and 0.99 W/m^2^)	TUNEL assay	Yes	[46]
***M. aeruginosa* FACH905**	Allelopathic submerged macrophyte, *Myriophyllum spicatum*,	Caspase assay and gel electrophoresis	Yes	[52]
***M. aeruginosa***	Glyphosate (1–10 mg/L)	Phosphatidylserine apoptosis assay based flow cytometry	Yes	[50]
***M. aeruginosa* FACHB905 and *M. viridis* (FACHB1337)**	fenoxaprop-p-ethyl (1–10 mg/L)	Phosphatidylserine apoptosis assay	Yes	[49]
***M. aeruginosa* FACHB905**	Vitamin C (0.6 mM)	TEM, Caspase-3 assay, and Hoechst 33342/PI staining assay	Yes	[64]
***M. aeruginosa* TAIHU98**	Prodigiosin (20–50 µg/mL) from *Hahella* sp. KA22	TEM, gel electrophoresis, Annexin V assay based on flow cytometry	Yes	[45]
***M. aeruginosa* DIANCHI 905**	Polyphenolic allelochemical pyrogallic acid (14 mg/L)	TEM, gel electrophoresis, and Annexin V apoptosis assay	Yes	[51]
***M. aeruginosa FACHB905*,**	H_2_O_2_ (0.1–1.5 mM)	SEM, Caspase-3 assay, and Hoechst 33342 staining assay	Yes	[47]
***M. aeruginosa* LB-2385**	Mesohaline conditions (<7 ppt)	Caspase-3 assay	Yes	[63]
***M.viridis* FACHB979**	0.2 to 10 mg/L in glyphosate	Phosphatidylserine Apoptosis Assay Kit	Yes	[67]

* ROS—reactive oxygen species.
